# Subcutaneous Extended-Release Buprenorphine Use in Pregnancy

**DOI:** 10.1155/2020/3127676

**Published:** 2020-07-17

**Authors:** Craig V. Towers, Heather Deisher

**Affiliations:** Obstetrics and Gynecology, Maternal-Fetal Medicine, High Risk Obstetrical Consultants, Knoxville, Tennessee 37920, USA

## Abstract

**Background:**

Opioid use disorder (OUD) in pregnancy is managed by medication-assisted treatment. Sublingual buprenorphine is one option, but subcutaneous extended-release buprenorphine (Sublocade®) is an alternate form administered in monthly injections. Through an extensive literature search, we did not find any prior publication on the use of Sublocade in pregnancy.

**Case:**

Two patients with OUD switched from sublingual buprenorphine to Sublocade. One patient received a total of eight injections and then discovered she was pregnant. Based on ultrasound dating, the last 5 administrations occurred during her pregnancy. The second patient received 6 injections with the last occurring at the time of her last menstrual period. Both declined further injections, as well as oral buprenorphine. Serial urine drug screens remained positive for buprenorphine through delivery in both cases. Neither the mothers nor the neonates experienced withdrawal symptoms or adverse outcomes. No birth anomalies were found. *Discussion*. Though further research is needed regarding the use of Sublocade in pregnancy, it is likely that other pregnancies will occur during this treatment modality. If this long-acting form of buprenorphine medication is found to be safe, it might play a role in managing some pregnant patients with OUD.

## 1. Background

Opioid use disorder (OUD) in pregnancy is a rising epidemic, especially in the United States. The Centers for Disease Control and Prevention reported that the prevalence of OUD more than quadrupled in the United States from 1999 to 2014 (from 1.5/1000 deliveries to 6.5/1000) [1]. The mainstay of medical management is to place these patients on medications for opioid use disorder (MOUD) that use longer-acting opioid drugs such as methadone or buprenorphine [2–6]. These two medications are administered in daily oral/sublingual dosages. Buprenorphine is also available in a subcutaneous extended-release form called Sublocade® [7]. Through a literature search of PubMed, Embase, Cochrane, Google Scholar, and Scopus, we did not find any prior publication on the use of Sublocade in pregnancy.

The objective of this report is to describe the pregnancy and delivery outcome of two patients who were receiving monthly Sublocade injections and then became pregnant.

## 2. Case Presentation

Patient number one had an 8-year history of opioid use disorder. She was being managed with sublingual buprenorphine and then chose to transition to monthly Sublocade injections; the first of which was administered about 3 months prior to her last menstrual period. The first two injections were 300 mg doses followed by monthly 100 mg doses. The last injection occurred at a gestational age of 17^4/7^ weeks. A total of 8 injections had been given (2-300 mg and 6-100 mg). At 18^3/7^ weeks' gestation, the patient discovered she was pregnant (unplanned) and declined any further injections. Oral buprenorphine was offered for continued MOUD during her pregnancy, but she wanted to be opioid-free at delivery and again declined.

Patient number two had a 6-year history of opioid use disorder. She was also being managed with sublingual buprenorphine but was transitioned to monthly Sublocade injections. She received a total of 6 injections (2-300 mg and 4-100 mg). The last injection occurred at the time of her last menstrual period. Oral buprenorphine was again offered for continued MOUD during her pregnancy, but she also declined. The prenatal care of both patients included an intensive behavioral health component.

Urine drug screens were performed throughout both pregnancies, and the buprenorphine and norbuprenorphine levels are seen in Table 1 for patient one and in Table 2 for patient two. Liver enzymes were checked and were normal in both cases. Urine drug screens were negative for any other drugs except buprenorphine. For both patients, subcutaneous nodules were palpable across the upper abdomen area (the location of the injection sites). These disappeared at around 34 weeks' gestation for patient number one and at 22 weeks' gestation for patient number two. Both patients delivered vaginally at term without complications. Both newborns had normal Apgar scores and were discharged with their mothers. No birth anomalies were identified. Both mothers required only acetaminophen and ibuprofen during the postpartum period for pain control.

Neither patient experienced any symptoms of opioid withdrawal. Both infants were monitored closely for 5 days postdelivery for signs of neonatal abstinence syndrome (NAS). NAS did not develop in either case. Patient number one was seen 8 weeks postdelivery, and her urine drug screen was still positive for buprenorphine and norbuprenorphine but at very low levels. She was doing well with no signs of opioid withdrawal. Patient number two was seen 6 weeks postdelivery, and her urine drug screen was negative. Both patients were continuing in intensive behavioral health, and the plan was to continue this for a minimum of 12 months postpartum. Both declined restarting any form of MOUD. Both newborns at follow-up (8 months for newborn one and 2 months for newborn two) were evaluated and had normal examinations and were progressing with normal milestones.

## 3. Discussion

The primary recommended treatment approach in managing opioid addiction is MOUD. A recurring problem in treating the OUD patient population is noncompliance with therapy leading to relapse or continued use of illicit opioid drugs, as well as drug diversion. This long-lasting injectable form of buprenorphine needs to be further studied to see if it has the potential benefit of preventing or limiting noncompliance as well as drug diversion.

Through our literature search, Sublocade use in pregnancy has not been previously reported. In these 2 cases, both women were receiving outpatient treatment for OUD with Sublocade injections. However, their outpatient management did not include contraception counseling despite being sexually active females of reproductive age. Both had unplanned pregnancies, desired to discontinue their Sublocade injections, and declined oral buprenorphine treatment. Urine drug levels for buprenorphine and the primary metabolite norbuprenorphine were serially monitored and consistently stayed positive for up to 30 weeks after the last injection for patient one and for 37 weeks in patient two. These also remained positive after the palpable subcutaneous injection site nodules resolved (13 weeks for patient one and 15 weeks for patient two). Both patients never experienced any symptoms of withdrawal, and both had uncomplicated pregnancies and deliveries. Neither newborn developed signs of NAS, and no anomalies were found.

Though this report only discusses two patients, the lack of withdrawal symptoms needs further discussion. These patients' urine drug levels slowly tapered over time to very low levels. This raises further questions and potential concerns. Are these lower levels still effective in preventing cravings and possible relapse or do they increase the possibility of relapse? This potential risk needs to be closely monitored in anyone who discontinues using the injectable form of buprenorphine and does not supplement their MOUD with another form of therapy. Additionally, many urine drug screens for buprenorphine have lower limits of detection and the levels for patient two could report out negative starting at 25 weeks' gestation and beyond, even though she was still positive.

Subcutaneous buprenorphine (Sublocade) was FDA approved in 2017 for the treatment of OUD. It is a monthly injection that can be used in place of daily oral buprenorphine [7]. Sublocade is designed to release a controlled rate of buprenorphine over the span of one month. It is dosed every four weeks so that a steady serum level can be maintained [7]. Sublocade has not been recommended for use in pregnancy due to a lack of safety studies. Buprenorphine is recommended for use in pregnancy, and therefore, the concern with using Sublocade is primarily the delivery system (Atrigel®) that for Sublocade is a 50 : 50 mixture of poly (D, L-lactate-co-glycolide) and N-methyl-2-pyrrolidone [7]. Poly (D, L-lactate-co-glycolide) has a molecular weight of 66,000 to 107,000 g/mol and is unlikely to cross the human placenta. N-Methyl-2-pyrrolidone (NMP) has a molecular weight of 99 g/mol and more than likely would cross the placenta.

Human studies on NMP use in pregnancy are lacking. In pregnant rats, NMP exposure by inhalation at doses equivalent to that delivered by Sublocade resulted in an increase in preimplantation losses, reduced fetal body weight, and delayed ossification [8]. At doses 3 times the maximum human daily dose based on body surface area, an increase in malformations and resorptions was seen [9]. However, studies that evaluate the effect of a drug and/or chemical used during pregnancy in animals versus humans do not often correlate.

A limitation in this case report is that both patients were not continuously observed to verify that oral buprenorphine was not taken by either patient (illicitly) after their last injection. However, urine buprenorphine and norbuprenorphine levels were always low; no other drugs were ever found in the random urine toxicology screens; both were actively involved in intensive behavioral health; and both desired to be drug-free at delivery. Therefore, it is unlikely any other additional buprenorphine was consumed by either patient.

In conclusion, subcutaneous extended-release buprenorphine will more than likely increase in use in the treatment of patients with OUD. With the large number of women of childbearing age suffering from this disease, most likely, there will be an increase in the number of pregnancies seen in women receiving this form of MOUD. Therefore, management of this specific population should include contraceptive counseling. In addition, further reports on the use of subcutaneous buprenorphine in pregnancy are also needed to better evaluate safety. If no adverse events are found, using subcutaneous buprenorphine may become an additional form of MOUD in pregnancy.

## Figures and Tables

**Table 1:** Urine buprenorphine and norbuprenorphine levels for patient one obtained through gestation, at delivery, and 8 weeks postdelivery—the last administration of subcutaneous extended-release buprenorphine was at 17^4/7^ weeks' gestation.

Gestational age (weeks)	Buprenorphine level (ng/mg creatinine)	Norbuprenorphine level (ng/mg creatinine)
22^4/7^	111	313
25^5/7^	85	156
28^6/7^	90	155
32^6/7^	64	88
35^5/7^	67	101
37^5/7^	59	104
Delivery 39^3/7^	43	87
8 weeks postdelivery	11	24

**Table 2:** Urine buprenorphine and norbuprenorphine levels for patient two obtained through gestation, at delivery, and 6 weeks postdelivery—the last administration of subcutaneous extended-release buprenorphine was at the time of her last menstrual period.

Gestational age (weeks)	Buprenorphine level (ng/mg creatinine)	Norbuprenorphine level (ng/mg creatinine)
9^3/7^	73	46
14^2/7^	54	33
18^2/7^	19	19
25^2/7^	8	11
28^2/7^	3	6
30^2/7^	4	0
33^2/7^	5	0
Delivery 37^4/7^	1	0
6 weeks postdelivery	0	0
